# Mediating effects of social detachment in young and middle-aged stroke patients between stigma and ability to return to work

**DOI:** 10.3389/fpubh.2025.1474345

**Published:** 2025-01-17

**Authors:** Jinping Dong, Xueying Ding, Yating Wen, Shujie Sun, Jingliang Wu, Jianhua Wang, Yang Fan, Guojing Hu, Zhang Xin, Jiayin Li, Xiaoyan Sun

**Affiliations:** ^1^Medical College, Weifang University of Science and Technology, Weifang, China; ^2^Neurosurgery Department of Weifang People's Hospital, Weifang, China

**Keywords:** young and middle-aged, stroke, work ability, social Isolation, mediation model

## Abstract

**Objective:**

To investigate the mediating effect of social isolation on the relationship between stigma and work ability in young and middle-aged stroke patients.

**Methods:**

From May 2022 to May 2024, a survey was conducted on young and middle-aged stroke patients visiting the neurology department of a tertiary hospital in Weifang. The survey utilized the General Information Questionnaire, the Rankin Scale, the General Isolation Scale, the Stigma Scale for Stroke Patients, and the Work Ability Scale.

**Results:**

The scores for work ability, social isolation, and stigma among young and middle-aged stroke patients were 44.23 ± 12.72, 46.27 ± 5.17, and 43.37 ± 4.48, respectively. There was a positive correlation between stigma and social isolation scores (*r* = 0.550, *P* < 0.01), and a negative correlation between stigma and work ability scores (*r* = −0.371, *P* < 0.01). Social isolation was negatively correlated with work ability (*r* = −0.418, *P* < 0.01). Multiple linear stepwise regression analysis indicated that stroke type, duration of illness, mRS score, stigma, and social isolation are significant factors affecting work ability in young and middle-aged stroke patients. Social isolation significantly mediated the relationship between stigma and work ability, accounting for 21.66% of the total effect.

**Conclusion:**

Stigma and social isolation can directly predict the level of work ability in young and middle-aged stroke patients. Additionally, stigma can indirectly influence work ability through social isolation.

## Introduction

Stroke is a group of acute cerebrovascular diseases caused by various factors. It is characterized by high incidence, high disability, and high mortality rates. Research indicates that the incidence rate of stroke in China is as high as 1.4%, and it is increasing at an annual rate of 8.3% (1). In recent years, with the improvement in treatment levels, the mortality rate of stroke patients has decreased. However, due to the high disability rate, survivors often have to endure a prolonged recovery period, suffering from irreversible bodily damage such as cognitive impairments, functional disabilities, and poor prognoses (2). Moreover, due to the sequelae of stroke, patients in the recovery phase often experience changes in behavioral abilities, social and familial roles, and interpersonal relationships. These changes can lead to psychological and emotional disorders, such as anxiety and depression, resulting in social avoidance, withdrawal, and anxiety—manifestations collectively described as social isolation (3). Patients with stronger stigma perceptions during the stroke recovery period tend to exhibit more negative self-concepts. As the self-concept represents the core of personality functioning, a more negative self-concept indicates poorer role adaptation and social adjustment, which further exacerbates social isolation (4). Studies have shown that stigma, as an emotional response of shame associated with having suffered a stroke, fundamentally reflects a negative coping strategy toward the condition. The stronger the stigma, the greater the impact on the patient's ability to return to work and reintegrate into society (5).

Research conducted in Sweden, the United States, the United Kingdom, and Japan has extensively explored the return-to-work outcomes of stroke patients, with increasing attention paid to related intervention studies. However, a search of the China National Knowledge Infrastructure (CNKI), Wanfang, and VIP databases revealed that domestic studies on the return-to-work outcomes of stroke patients began around 2,000, with limited publications to date (6). Most existing research in China focuses on the isolated effects of stigma and social isolation on the return-to-work abilities of young and middle-aged stroke patients. However, the mechanisms underlying the interactions among stigma, social isolation, and return-to-work ability remain unclear. The theory of unpleasant symptoms provides a framework for describing the influencing factors of symptom experiences and clarifies the relationships among these factors. This study adopts the theory of unpleasant symptoms to hypothesize that social isolation may act as a key mediator between stigma and return-to-work ability in young and middle-aged stroke patients (7). The findings aim to inform the development of vocational rehabilitation strategies tailored to the needs of Chinese stroke patients in the recovery phase, facilitating their successful reintegration into the workforce.

## Materials and methods

### Study subjects

The study selected stroke recovery patients who underwent follow-up examinations at Weifang City Affiliated Hospital from May 2022 to May 2024. Inclusion criteria were: (1) The young and middle-aged stroke population refers to patients clinically diagnosed with stroke who meet the World Health Organization (WHO) age criteria for young and middle-aged individuals, as well as the labor law's definition of working-age. This includes males aged 18–59 years and females aged 18–54 years. age between 18 and 60 (8); (2) meeting stroke diagnostic criteria; (3) regularly attending follow-up appointments as per medical advice after discharge; (4) having a certain level of education and ability to communicate; and (5) being informed and agreeing to participate in the study. Exclusion criteria were: (1) Severe motor aphasia, sensory aphasia, or mixed aphasia. (2) Being in the acute phase or having an unstable condition; (3) Having other severe systemic diseases; (4) Having psychiatric or psychological disorders.

The sample size was estimated using the cross-sectional study method, with the sample size being 10–20 times the number of study variables. This study included 11 variables and accounted for a 10% invalid questionnaire rate. The calculated sample size range was 130–240, and 234 patients were ultimately included in the study. General information is detailed in Table 1.

**Table 1:** Comparison of total scores for return to work ability among patients with different demographic and disease characteristics (*n* = 234).

**Variable**	**Classification**	**Sample size**	**Score (M ±SD)**	***t/F***	***P***
**Sex**					
	Male	128	40.25 ± 10.73	0.357	0.536
	Female	106	39.25 ± 7.32		
**Age**					
	< 30	46	34.00 ± 9.41	3.102	< 0.05
	35~45	110	44.06 ± 7.31		
	46~59	78	33.23 ± 7.42		
**Education**					
	Primary school	47	39.13 ± 6.23	2.377	0.058
	Junior high school	88	40.06 ± 11.72		
	High school or technical school	63	39.42 ± 10.78		
	College or higher	36	44.58 ± 7.98		
**Marital status**					
	Married	192	44.23 ± 8.97	2.023	0.087
	Unmarried	33	44.58 ± 12.15		
	Divorced	7	39.00 ± 10.10		
	Widowed	2	20.50 ± 8.90		
**Family income**					
	< 1,000	16	39.84 ± 7.63	2.278	0.082
	1,000~3,000	21	39.45 ± 11.13		
	3,000~5,000	105	40.05 ± 13.45		
	>5,000	92	44.91 ± 12.07		
**Stroke type**					
	Ischemic stroke	122	43.21 ± 14.87	6.723	< 0.01
	Hemorrhagic stroke	74	39.65 ± 14.08		
	Mixed type	38	36.73 ± 13.24		
**Duration of illness (months)**					
	1~3	176	40.24 ± 10.91	5.617	< 0.01
	3~6	24	34.78 ± 10.23		
	6~12	16	37.23 ± 7.59		
	>12	18	44.11 ± 4.72		
**Number of stroke occurrences**					
	1	167	44.22 ± 4.73	3.460	< 0.05
	2	46	41.27 ± 18.32		
	≥3	21	38.69 ± 22.72		
**Medical payment method**					
	Urban employee basic medical insurance	125	40.29 ± 11.64	1.098	0.351
	Urban and rural resident basic medical insurance	83	40.17 ± 12.75		
	Commercial medical insurance	24	33.40 ± 12.45		
	Other	2	40.12 ± 7.44		
**mRS score**					
	1	37	36.62 ± 13.73	15.725	< 0.001
	2	81	36.69 ± 13.96		
	3	85	46.25 ± 15.2		
	4	31	33.74 ± 10.94		

### Research tools

#### General information questionnaire

Designed by the researchers and team members based on a literature review and research objectives. It includes questions on gender, age, educational level, marital status, employment status, household income, type of stroke, duration of illness, number of stroke occurrences, method of medical payment, current employment status, and mRS score.

Modified Rankin Scale (mRS) The Modified Rankin Scale (mRS) is used to evaluate neurological recovery after a stroke. The total score ranges from 0 to 6, with higher scores indicating more severe disability: 0 represents no disability, 5 represents severe disability, and 6 represents death. The mRS (disability level) is a commonly used scale for assessing functional outcomes after a stroke. It has a Cronbach's alpha coefficient of 0.78 and a test-retest reliability of 0.81 (9).

#### General alienation scale (GAS)

This scale was developed by Jessor et al. (10) and translated into Chinese by Wu Shuang et al. (11) to assess the social isolation of participants. The scale includes 15 items across four dimensions: self-alienation (3 items), alienation from others (5 items), mistrust (4 items), and meaninglessness (3 items). It uses a 4-point Likert scale, ranging from “strongly disagree” to “strongly agree,” scoring from 1 to 4 points, respectively. The total score ranges from 15 to 60 points, with higher scores indicating a greater degree of social isolation. The scale has good reliability with a Cronbach's alpha coefficient of 0.770. It has been used by scholars to measure social isolation in middle-aged and young stroke patients, with a Cronbach's alpha coefficient of 0.807. In this study, the Cronbach's alpha coefficient of the scale is 0.829.

#### Stroke patient stigma scale

This scale was developed by Zhu Minfang et al. (12) to assess stigma in stroke patients. It includes 16 items across four dimensions: physical disability (4 items), self-perception (5 items), experiences of discrimination (4 items), and social interaction (3 items). The scale uses a 5-point Likert scale, ranging from “never” to “always,” scored from 1 to 5 points, respectively. The total score ranges from 16 to 80 points, with higher scores indicating stronger stigma. The scale has a Cronbach's alpha coefficient of 0.916. It has been evaluated in stroke patients, with a Cronbach's alpha coefficient of 0.924. In this study, the Cronbach's alpha coefficient of the scale is 0.918.

#### Work-ability support scale (WSS)

This scale was developed by Turner-Stokes et al. (13) and translated into Chinese by Guo Yawen et al. (14) to assess the level of work-ability support in patients. The scale includes 16 items across three dimensions: physical (5 items), cognitive and communication (5 items), and social (6 items). It uses a 7-point Likert scale, ranging from “continuous support” to “independent work without improvement,” scored from 1 to 7 points, respectively. The total score ranges from 16 to 112 points, with higher scores indicating a stronger ability to return to work. The scale has a Cronbach's alpha coefficient of 0.932. In this study, the Cronbach's alpha coefficient of the scale is 0.856.

### Data collection method

This study employed a questionnaire survey method. Trained investigators distributed paper-based questionnaires. Initially, the investigators explained the research purpose and significance to the participants using standardized instructions and obtained informed consent before distributing the questionnaires. Participants completed the questionnaires independently. For patients lacking writing or reading abilities, investigators read each item aloud and objectively recorded the responses on the spot. After completion, the investigators collected and immediately checked the questionnaires. If any items were missed or errors were found, the investigators clarified with the participants, made corrections, and confirmed accuracy before final collection. A total of 250 questionnaires were distributed, resulting in 234 valid responses, yielding an effective response rate of 93.6%.

### Statistical methods

Data were entered into Excel by two individuals to ensure accuracy and logical consistency. After verification, SPSS 22.0 and AMOS 21.0 software were used for data analysis. Measurement data conforming to a normal distribution were described using mean ± standard deviation. Count data were described using frequencies and percentages. Data were analyzed using independent sample *t*-tests, one-way ANOVA, Pearson correlation analysis, and multiple linear regression analysis. Based on the results of the Pearson correlation analysis, a path relationship model was established and fitted using the maximum likelihood method, with subsequent model modifications. The significance level was set at α = 0.05.

## Results

Comparison of Total Work-ability Scores Among Patients with Different Demographic and Disease Characteristics. There were statistically significant differences (*P* < 0.05) in the total work-ability scores among middle-aged and young stroke patients when comparing different ages, stroke types, illness durations, stroke occurrences, and mRS scores. Differences in other factors were not statistically significant. For detailed information, see Table 1.

Scores of Stigma, Social Isolation, and Work-ability Among Middle-aged and Young Stroke Patients. In this study, the average score for social isolation among middle-aged and young stroke patients was 46.27 ± 5.17, indicating a relatively high level, with 66.3% of patients scoring above the average. The average stigma score was 43.37 ± 4.48, with 46.16% of participants exhibiting moderately high levels. The average score for work-ability was 44.23 ± 12.72, indicating a moderately low level. For detailed information, see Table 2.

**Table 2:** Scores of disease shame, social isolation, and return to work ability among young and middle-aged stroke patients (*n* = 234).

**Item**	**Dimension score**	**Item average score**
Disease shame	43.37 ± 4.48	2.71 ± 0.28
Physical disabilities (4)	8.84 ± 2.35	2.21 ± 0.59
Self-perception (5)	14.43 ± 4.21	2.89 ± 0.84
Discrimination experience (4)	11.68 ± 2.57	2.92 ± 0.64
Social interaction (3)	8.62 ± 2.72	2.87 ± 0.91
Social isolation	46.27 ± 5.17	3.08 ± 0.27
Others' alienation (5)	16.72 ± 2.32	3.34 ± 0.46
Self-alienation (3)	9.43 ± 1.19	3.14 ± 0.40
Sense of meaninglessness (3)	10.16 ± 1.92	3.38 ± 0.64
Suspicion (4)	13.71 ± 1.82	3.42 ± 0.46
Return to work ability	44.23 ± 12.72	2.76 ± 0.80
Physiology (5)	11.32 ± 6.08	2.26 ± 1.22
Thinking and communication (5)	10.53 ± 7.75	2.10 ± 1.55
Social behavior (6)	12.34 ± 8.94	2.05 ± 1.49

Correlation Analysis of Stigma, Social Isolation, and Work-ability Among Middle-aged and Young Stroke Patients. Stigma was positively correlated with social isolation scores (*r* = 0.5496, *P* < 0.01) and negatively correlated with work-ability scores (*r* = −0.371, *P* < 0.01). Social isolation was negatively correlated with work-ability scores (*r* = −0.418, *P* < 0.01).

Regression Analysis of Return-to-Work Ability Among Middle-aged and Young Stroke Patients. Multiple linear stepwise regression analysis was conducted with return-to-work ability of middle-aged and young stroke patients as the dependent variable. Independent variables included age, stroke type, stroke occurrences, duration of illness, mRS score, stigma, and social isolation scores. Categorical variable assignments are detailed in Table 3. The results indicated that stroke type, duration of illness, mRS score, stigma, and social isolation all entered the regression equation (*P* < 0.05), as shown in Table 4.

**Table 3:** Independent variable assignments.

**Independent variable**	**Assignment**
Age (years)	< 30 = 1; 30–45 = 2; 46–59 = 3
Number of strokes	1 = 1; 2 = 2; ≥3 = 3
Type of stroke	Ischemic stroke = 1; Hemorrhagic stroke = 2; Mixed type = 3
Duration of illness	1–3 months = 1; 3–6 months = 2; 6–12 months = 3; >12 months = 4
mRS score	1 = 1; 2 = 2; 3 = 3; 4 = 4

**Table 4:** Multiple linear regression analysis of factors influencing return to work ability among young and middle-aged stroke patients (*n* = 234).

**Item**	**Regression coefficient**	**Standard error**	**Standardized coefficient**	***t*-value**	***p*-value**
(Constant)	41.45	2.178	-	13.63	0.001
Stroke type	2.006	0.854	0.132	2.179	0.017
Duration of illness	2.236	1.178	0.097	1.927	0.026
mRS score	−1.476	0.235	−0.186	−5.802	0.001
Disease shame	−0.269	0.041	−0.289	−7.341	0.001
Social isolation	−0.478	0.039	−0.443	−10.782	< 0.001

*R*^2^ = 0.612, Adjusted *R*^2^ = 0.601, *F* = 135.703, *p* ≤ 0.001.

**Figure 1:**
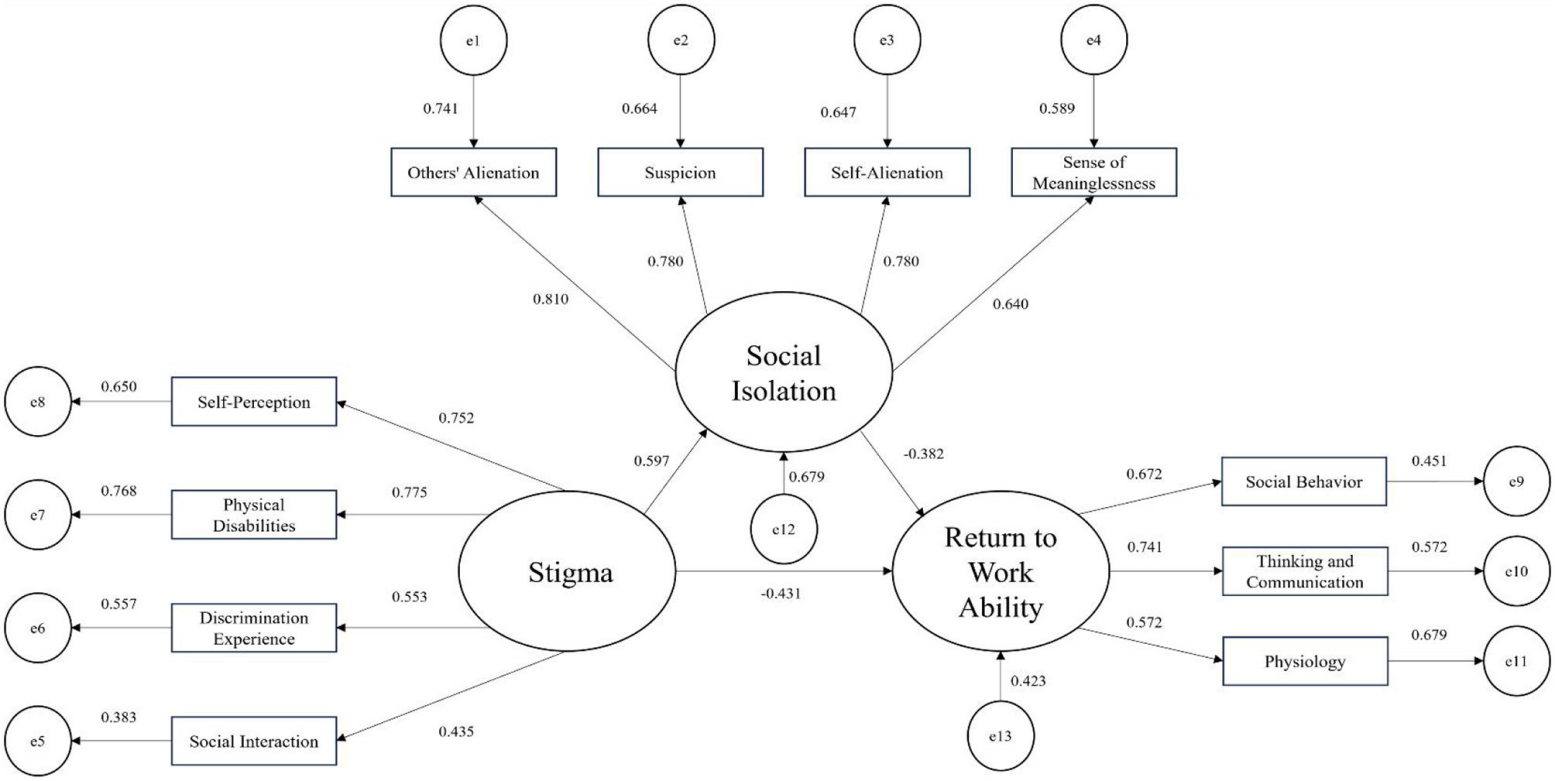
Pathways of the influence of disease shame and social isolation on return to work ability among young and middle-aged stroke patients.

Structural Equation Model of Return-to-Work Ability Among Middle-aged and Young Stroke Patients. A structural equation model was constructed with stigma as the independent variable, return-to-work ability as the dependent variable, and social isolation as the mediating variable. The model was fitted using the maximum likelihood method, and the parameters indicated good model fit. Based on the correlation analysis and multiple linear stepwise regression results, AMOS 21.0 was used to establish the hypothetical model and conduct path analysis. The model was tested and modified according to modification indices, resulting in a structural equation model where emotional expression conflict is the independent variable and fear of disease progression acts as the mediating variable, collectively influencing social isolation. The detailed model is shown in Figure 1. The fit indices for the structural equation model were as follows: χ^2^/df = 1.685, RMSEA = 0.054 (< 0.08), CFI = 0.965, NFI = 0.920 IFI = 0.966 GFI = 0.946 AGFI = 0.914.

We employed bias-corrected non-parametric percentile Bootstrap analysis, with 5,000 iterations of resampling and a 95% confidence interval, to test the significance of the mediating effect. The results indicated the following: (1) Stigma significantly positively predicts social isolation (*B* = 0.591, *P* < 0.001); (2) Stigma negatively predicts return-to-work ability (*B* = −0.431, *P* < 0.001); (3) Social isolation directly negatively predicts return-to-work ability (*B* = −0.382, *P* < 0.001); (4) Stigma indirectly affects return-to-work ability through social isolation (*B* = 0.165, *P* < 0.001), suggesting that social isolation partially mediates the relationship between stigma and return-to-work ability, accounting for 21.66% of the total effect (0.165/0.762). Detailed results are presented in Table 5.

**Table 5:** Effect scores of fear of disease progression as a mediating variable influencing social isolation through emotional expression conflict (*n* = 282).

**Effect**	**Coefficient B**	**SE**	***t*-value**	***p*-value**	**95% CI**
Total effect	0.762	0.065	10.063	0.001	0.435–0.641
Direct effect	0.597	0.088	4.215	0.001	0.170–0.472
Indirect effect	0.165	0.047	2.156	0.001	0.130–0.391

## Discussion

The results of this study show that the return-to-work ability level of stroke patients in the recovery period scores at (44.23 ± 12.72), which is below average. This finding is consistent with the research results of G. Yawen, indicating that the issue of return-to-work ability in young and middle-aged stroke patients needs to be a focus for healthcare professionals (14). Possible reasons include that young and middle-aged individuals often shoulder multiple family and social roles. However, due to the diagnosis of the disease and the associated stigma of stroke, their enthusiasm for participating in social activities diminishes. Additionally, postoperative physical disabilities and changes in appearance cause psychological distress and a decrease in self-esteem among these patients. Consequently, they lack confidence in social interactions and are reluctant to actively engage in activities (15). The results of multiple linear regression analysis indicate that stroke type, duration of illness, and disability severity (mRS score) are the primary influencing factors on the return-to-work ability. Rooman et al. study results indicate that patients with an mRS score of 0–1 (indicating mild disability) have the ability to return to work, thereby suggesting a higher likelihood of returning to work (16). Physical function is just one component of work ability, but remains an important factor to consider when assessing return-to-work capabilities, particularly in ensuring individuals can meet job requirements safely. Additionally, stroke type can also impact a patient's ability to return to work, possibly due to the severity of the condition and prognosis. A study from Japan noted that patients leaving work due to hemorrhagic stroke may require longer periods to fully return to their job compared to those with ischemic stroke, aligning with findings in this study (17). This study also indicated that the differences in return-to-work ability scores among young and middle-aged stroke patients with varying lengths of illness were statistically significant. This may be related to patients experiencing a prolonged rehabilitation period leading to better physical recovery and higher return-to-work ability levels. Healthcare providers can plan vocational rehabilitation assessments and interventions early in the patient's recovery based on their condition and progress. Providing relevant work-related information in a timely manner can enhance their confidence in recovery and ability to return to work.

The results of this study indicate a negative correlation between stigma and return-to-work ability in young and middle-aged stroke patients (*r* = −0.371, *P* < 0.01). Specifically, higher levels of stigma were associated with lower return-to-work scores. In this study, 46.16% of patients exhibited high levels of stigma, with a mean return-to-work score of 49.41 ± 7.17. These findings align with previous research, confirming that higher stigma levels result in diminished return-to-work ability (18). The study also revealed a positive correlation between stigma and social isolation (*r* = 0.5496, *P* < 0.01), consistent with findings from related research. Young and middle-aged stroke patients with lower levels of physical activity reported higher social isolation and stigma, suggesting that elevated stigma is a major barrier to mobility. Furthermore, this study identified a negative correlation between social isolation and return-to-work ability (*r* = −0.418, *P* < 0.01), indicating that higher levels of social isolation were associated with lower return-to-work scores. Among the participants, 66.3% experienced social isolation, with a mean return-to-work score of 56.78 ± 12.02, highlighting significant limitations in return-to-work ability. The strong association between social isolation and reduced return-to-work capacity aligns with findings from prior studies (19). The underlying reason may be that social isolation leads to discomfort in social interactions, thereby reducing patients' willingness and capacity to engage in work environments. Additionally, a lack of social support may exacerbate this isolation, as patients often lack the encouragement and assistance necessary to re-enter the workforce. Other studies have also reported a significant relationship between social isolation and physical activity limitations, which may further restrict workplace participation (20). Therefore, providing increased social support and psychological interventions for young and middle-aged stroke patients could help alleviate social isolation, ultimately improving their ability to return to work.

The results of this study demonstrate that social isolation partially mediates the relationship between stigma and return-to-work ability in young and middle-aged stroke patients, with an effect value of 0.165, accounting for 21.66% of the total effect. This finding indicates that stigma can indirectly influence return-to-work ability through social isolation. Consistent with this result, Yandi et al. (21) found that lower stigma levels predict reduced social isolation in stroke patients. The possible explanation is that after experiencing a stroke, patients may feel shame and embarrassment due to communication difficulties, such as language impairments (22). This may lead to social withdrawal and a reluctance to engage with others. The lack of effective communication can result in behaviors characterized by social isolation, which in turn reduces motivation for productive activities and daily living, ultimately limiting return-to-work ability (23). Research has shown that as age increases and physical function declines, social isolation and stigma intensify in young and middle-aged stroke patients. This exacerbates functional limitations in daily activities, further reducing their likelihood of re-entering the workforce and negatively affecting their productive capacity and quality of life (24). Nafei highlighted that young and middle-aged patients often express a strong desire to return to work (25). However, concerns about potential threats to their self-esteem significantly hinder their recovery of work capacity, leading to a higher prevalence of social isolation. These findings suggest that frontline healthcare providers could target social isolation as a key intervention point. Strategies may include reducing stigma, minimizing sedentary behavior, and encouraging participation in social activities and outdoor exercise (26). Such interventions could enhance patients' return-to-work ability and improve their overall recovery outcomes.

### Limitation

The limitations of this study include the selection of a single research center, the use of convenience sampling, and a cross-sectional study design, which may restrict the generalizability of the findings. The external validity of the mediation model requires further confirmation. Future studies should involve multi-center, large-sample, and prospective designs, extending research settings to include elder care facilities and diverse regions. Additionally, further exploration of other potential variables and their underlying mechanisms is warranted.

## Conclusion

Stigma and social isolation are independent influencing factors on return-to-work ability in young and middle-aged stroke patients. Stigma can negatively predict return-to-work ability both directly and indirectly through social isolation. Social isolation plays a partial mediating role in the relationship between stigma and return-to-work ability. Healthcare providers and managers should prioritize interventions aimed at reducing stigma and modifying habitual social isolation behaviors. It is recommended to implement intervention strategies based on pathways influencing social isolation. Nursing managers can adopt various approaches, including supportive-expressive group therapy, enhanced health education, personalized care plans, narrative therapy, mindfulness-based interventions, progressive focused interviews, and peer support interventions, to reduce social isolation among stroke patients in the recovery phase. These measures aim to enhance return-to-work ability and improve physical activity and quality of life in young and middle-aged stroke patients.

## Data Availability

The original contributions presented in the study are included in the article/Supplementary material, further inquiries can be directed to the corresponding authors.

## References

[1] Yongping L Juan W Shuhui D Zhenzhen W Yan H. Study on the social isolation status and influencing factors of stroke recovery patients. Nurs Manage J. (2021) 21:685–91.

[2] Hongbin Z. Study on the Self-management Status and Influencing Factors of Young and Middle-aged Stroke Patients. Shandong University of Traditional Chinese Medicine (2021).

[3] Dan J Wei L Ning G Jiaqi Z Jianan L Fengjuan Z. Study on the correlation between fall efficacy and social support in elderly stroke patients. Nurs J. (2020) 27:65–7.

[4] Wenjuan Y Zhuangmiao L Mengting Y Sheen L Yanhong C. A systematic review of influencing factors of stigma in stroke patients. J Nurs. (2022) 29:46–52.

[5] Jun Z Yan G Guangmei L. Status quo and influencing factors of social alienation in patients with sequelae stage of ischemic stroke. Prevent Med South China. (2022) 48:854–6+860.

[6] Cong T Chengping Q Chen J Lei Z Guangyue Y Min Y. A systematic evaluation of the status quo and influencing factors of cancer population returning to work in China. Milit Nurs. (2022) 39:73–7.

[7] Blakeman JR. An integrative review of the theory of un-J. J Adv Nurs Pleasant Sympt. (2019) 75:946–61. 10.1111/jan.13906 30397941

[8] Chinese Society of Neurology. Cerebrovascular disease group of the chinese society of neuroloy. Chin J Neurol. (2019) 52:710–5.

[9] Banks JL Marotta CA. Outcomes validity and reliability of the modified Rankin scale: implications for stroke clinical trials: a literature review and synthesis. Stroke. (2007) 38:1091–6. 10.1161/01.STR.0000258355.23810.c6 17272767

[10] Jessor R. Problem-behavior theory, psychosocial development, and adolescent problem drinking. Br J Addict. (1987) 82:331–42. 10.1111/j.1360-0443.1987.tb01490.x 3472582

[11] Shuang W Yanzhang L Xiaolin Z Qi D Ling D Siyi G. Reliability and validity analysis of the general alienation scale in the elderly. J Chengdu Med College. (2015) 10:751–4.

[12] Minfang Zhu HZ Deng Y Wang X Yang L Li M. The development and validation of the stroke patients' disease shame scale. J Nurs Sci. (2019) 34:70–3.27409075

[13] Turner-Stokes L Fadyl J Rose H Williams H Schlüter P McPherson K. The work-ability support scale: evaluation of scoring accuracy and rater reliability. J Occup Rehabil. (2014) 24:511–24. 10.1007/s10926-013-9486-1 24338285 PMC4118042

[14] Yawen G. Translation and Application Research of the Work Ability Support Scale in Reemployment of Middle-aged and Young Stroke Patients. Zhengzhou University (2020).

[15] Menglu Z. Investigation and Construction of Prediction Model of Return to Work Readiness and Maintenance in Young and Middle-Aged Stroke Patients. Qingdao University (2021).

[16] Minhua L. Research progress of psychological status analysis and intervention measures of breast cancer patients after surgery. Chin Contemp Med. (2015) 22:16–20.

[17] Rooman C Sterkens P Schelfhout S Van Royen A Baert S Derous E. Successful return to work after burnout: an evaluation of job, person- and private-related burnout determinants as determinants of return-to-work quality after sick leave for burnout. Disabil Rehabil. (2022) 44:7106–15. 10.1080/09638288.2021.1982025 34607496

[18] Endo M Sairenchi T Kojimahara N Haruyama Y Sato Y Kato R. Sickness absence and return to work among Japanese stroke survivors: a 365-day cohort study. BMJ Open. (2016) 6:e009682. 10.1136/bmjopen-2015-009682 26729388 PMC4716216

[19] Chen J Li S. Clinical study of neurology nursing on cerebral apoplexy rehabilitation. Transl Neurosci. (2019) 10:164–7. 10.1515/tnsci-2019-0029 31410298 PMC6689212

[20] Jie N Juan C Wenjing S Yuanyuan L Hongli L Gaozhen Y. A study on the correlation between the level of stigma and the quality of life of patients after breast cancer surgery in two hospitals in Shandong Province. Med Soc. (2020) 33:81–4.

[21] Yandi Z Ronghui L Feng H. The effect of stigma on work behavior in breast cancer survivors returning to work. J Nurs Sci. (2020) 35:67–70.

[22] Lili L Lijun W Chunge D Yongli W Zhenxiang Z. Research status and prospect of social participation in middle-aged and young stroke patients. J Nurs Administ. (2018) 18:732–6.

[23] Li M Zhu WJ Luo Q Chen H Duan Y Xie HZ. Psychological experience of humanistic care among medical staff in stroke wards: a qualitative research study conducted in China. Front Psychiatry. (2022) 13:791993. 10.3389/fpsyt.2022.791993 35401272 PMC8989731

[24] Yeo SN Zainal H Tang CS Tong EM Ho CS Ho RC. Success/failure condition influences attribution of control, negative affect, and shame among patients with depression in Singapore. BMC Psychiatry. (2017) 17:285. 10.1186/s12888-017-1451-7 28768488 PMC5541725

[25] Nafei X. Descriptive Nature of Fear Experience in Stroke Patients. Zhejiang Chinese Medicine University (2021).

[26] Ford ME Geurtsen GJ Groet E Van Bennekom CAM Van Someren EJW. A blended eHealth intervention for insomnia following acquired brain injury: study protocol for a randomized controlled trial. Trials. (2020) 21:861. 10.1186/s13063-020-04789-y 33066812 PMC7566121

