# Supplementary material for: Mediating effects of social detachment in young and middle-aged stroke patients between stigma and ability to return to work
Source: Front Public Health. 2025 Jan 17;13:1474345. doi: 10.3389/fpubh.2025.1474345 (PMC11782025; doi:10.3389/fpubh.2025.1474345)
Supplement: Supplementary file 1 [file Table_1.docx]

##### Supplementary Material 1

##### Modification Indices (Group number 1 - Default model)

##### Covariances: (Group number 1 - Default model)

|  |  |  | M.I. | Par Change |
| --- | --- | --- | --- | --- |
| e8 | <--> | e12 | 5.449 | -.081 |
| e6 | <--> | e13 | 6.507 | -.116 |
| e6 | <--> | e11 | 4.308 | -.100 |
| e6 | <--> | e9 | 4.036 | -.109 |
| e5 | <--> | e12 | 4.456 | .104 |
| e4 | <--> | e13 | 6.941 | .118 |
| e3 | <--> | e8 | 4.892 | .074 |
| e3 | <--> | e6 | 4.989 | -.093 |
| e2 | <--> | e13 | 7.261 | -.101 |

##### Variances: (Group number 1 - Default model)

|  |  |  | M.I. | Par Change |
| --- | --- | --- | --- | --- |

##### Regression Weights: (Group number 1 - Default model)

|  |  |  | M.I. | Par Change |
| --- | --- | --- | --- | --- |
| 自我感受 | <--- | 无意义感 | 4.608 | -.092 |
| 自我感受 | <--- | 他人疏离感 | 5.099 | -.109 |
| 受歧视经历 | <--- | 生理 | 6.396 | -.157 |
| 受歧视经历 | <--- | 社交与行为 | 5.922 | -.128 |
| 社会交往 | <--- | 无意义感 | 4.609 | .128 |
| 无意义感 | <--- | 重返工作能力 | 5.019 | .202 |
| 无意义感 | <--- | 社交与行为 | 5.910 | .126 |
| 自我疏离感 | <--- | 自我感受 | 4.422 | .105 |
| 怀疑感 | <--- | 生理 | 5.136 | -.116 |

##### Minimization History (Default model)

| Iteration |  | Negative eigenvalues | Condition # | Smallest eigenvalue | Diameter | F | NTries | Ratio |
| --- | --- | --- | --- | --- | --- | --- | --- | --- |
| 0 | e | 6 |  | -.449 | 9999.000 | 913.406 | 0 | 9999.000 |
| 1 | e | 3 |  | -.078 | 2.055 | 405.969 | 20 | .486 |
| 2 | e | 0 | 323.304 |  | 1.286 | 182.568 | 5 | .705 |
| 3 | e | 0 | 80.344 |  | .922 | 160.852 | 3 | .000 |
| 4 | e | 1 |  | -.014 | 1.002 | 106.296 | 1 | .688 |
| 5 | e | 0 | 65.542 |  | .651 | 74.337 | 7 | .955 |
| 6 | e | 0 | 162.342 |  | .325 | 69.797 | 1 | 1.140 |
| 7 | e | 0 | 315.802 |  | .249 | 69.160 | 1 | 1.124 |
| 8 | e | 0 | 456.818 |  | .088 | 69.083 | 1 | 1.082 |
| 9 | e | 0 | 492.233 |  | .020 | 69.082 | 1 | 1.018 |
| 10 | e | 0 | 489.320 |  | .001 | 69.082 | 1 | 1.001 |

##### Model Fit Summary

##### CMIN

| Model | NPAR | CMIN | DF | P | CMIN/DF |
| --- | --- | --- | --- | --- | --- |
| Default model | 25 | 69.082 | 41 | .004 | 1.685 |
| Saturated model | 66 | .000 | 0 |  |  |
| Independence model | 11 | 858.375 | 55 | .000 | 15.607 |

##### RMR, GFI

| Model | RMR | GFI | AGFI | PGFI |
| --- | --- | --- | --- | --- |
| Default model | .056 | .946 | .914 | .588 |
| Saturated model | .000 | 1.000 |  |  |
| Independence model | .306 | .461 | .354 | .384 |

##### Baseline Comparisons

| Model | NFI Delta1 | RFI rho1 | IFI Delta2 | TLI rho2 | CFI |
| --- | --- | --- | --- | --- | --- |
| Default model | .920 | .892 | .966 | .953 | .965 |
| Saturated model | 1.000 |  | 1.000 |  | 1.000 |
| Independence model | .000 | .000 | .000 | .000 | .000 |

##### Parsimony-Adjusted Measures

| Model | PRATIO | PNFI | PCFI |
| --- | --- | --- | --- |
| Default model | .745 | .685 | .719 |
| Saturated model | .000 | .000 | .000 |
| Independence model | 1.000 | .000 | .000 |

##### NCP

| Model | NCP | LO 90 | HI 90 |
| --- | --- | --- | --- |
| Default model | 28.082 | 9.000 | 55.040 |
| Saturated model | .000 | .000 | .000 |
| Independence model | 803.375 | 712.222 | 901.951 |

##### FMIN

| Model | FMIN | F0 | LO 90 | HI 90 |
| --- | --- | --- | --- | --- |
| Default model | .296 | .121 | .039 | .236 |
| Saturated model | .000 | .000 | .000 | .000 |
| Independence model | 3.684 | 3.448 | 3.057 | 3.871 |

##### RMSEA

| Model | RMSEA | LO 90 | HI 90 | PCLOSE |
| --- | --- | --- | --- | --- |
| Default model | .054 | .031 | .076 | .355 |
| Independence model | .250 | .236 | .265 | .000 |

##### AIC

| Model | AIC | BCC | BIC | CAIC |
| --- | --- | --- | --- | --- |
| Default model | 119.082 | 121.797 | 205.465 | 230.465 |
| Saturated model | 132.000 | 139.167 | 360.051 | 426.051 |
| Independence model | 880.375 | 881.569 | 918.383 | 929.383 |

##### ECVI

| Model | ECVI | LO 90 | HI 90 | MECVI |
| --- | --- | --- | --- | --- |
| Default model | .511 | .429 | .627 | .523 |
| Saturated model | .567 | .567 | .567 | .597 |
| Independence model | 3.778 | 3.387 | 4.202 | 3.784 |

##### HOELTER

| Model | HOELTER .05 | HOELTER .01 |
| --- | --- | --- |
| Default model | 193 | 220 |
| Independence model | 20 | 23 |

##### Execution time summary

| Minimization: | .016 |
| --- | --- |
| Miscellaneous: | .119 |
| Bootstrap: | .000 |
| Total: | .135 |

##### Model Fit Summary

##### CMIN

| Model | NPAR | CMIN | DF | P | CMIN/DF |
| --- | --- | --- | --- | --- | --- |
| Default model | 25 | 69.082 | 41 | .004 | 1.685 |
| Saturated model | 66 | .000 | 0 |  |  |
| Independence model | 11 | 858.375 | 55 | .000 | 15.607 |

##### RMR, GFI

| Model | RMR | GFI | AGFI | PGFI |
| --- | --- | --- | --- | --- |
| Default model | .056 | .946 | .914 | .588 |
| Saturated model | .000 | 1.000 |  |  |
| Independence model | .306 | .461 | .354 | .384 |

##### Baseline Comparisons

| Model | NFI Delta1 | RFI rho1 | IFI Delta2 | TLI rho2 | CFI |
| --- | --- | --- | --- | --- | --- |
| Default model | .920 | .892 | .966 | .953 | .965 |
| Saturated model | 1.000 |  | 1.000 |  | 1.000 |
| Independence model | .000 | .000 | .000 | .000 | .000 |

##### Parsimony-Adjusted Measures

| Model | PRATIO | PNFI | PCFI |
| --- | --- | --- | --- |
| Default model | .745 | .685 | .719 |
| Saturated model | .000 | .000 | .000 |
| Independence model | 1.000 | .000 | .000 |

##### NCP

| Model | NCP | LO 90 | HI 90 |
| --- | --- | --- | --- |
| Default model | 28.082 | 9.000 | 55.040 |
| Saturated model | .000 | .000 | .000 |
| Independence model | 803.375 | 712.222 | 901.951 |

##### FMIN

| Model | FMIN | F0 | LO 90 | HI 90 |
| --- | --- | --- | --- | --- |
| Default model | .296 | .121 | .039 | .236 |
| Saturated model | .000 | .000 | .000 | .000 |
| Independence model | 3.684 | 3.448 | 3.057 | 3.871 |

##### RMSEA

| Model | RMSEA | LO 90 | HI 90 | PCLOSE |
| --- | --- | --- | --- | --- |
| Default model | .054 | .031 | .076 | .355 |
| Independence model | .250 | .236 | .265 | .000 |

##### AIC

| Model | AIC | BCC | BIC | CAIC |
| --- | --- | --- | --- | --- |
| Default model | 119.082 | 121.797 | 205.465 | 230.465 |
| Saturated model | 132.000 | 139.167 | 360.051 | 426.051 |
| Independence model | 880.375 | 881.569 | 918.383 | 929.383 |

##### ECVI

| Model | ECVI | LO 90 | HI 90 | MECVI |
| --- | --- | --- | --- | --- |
| Default model | .511 | .429 | .627 | .523 |
| Saturated model | .567 | .567 | .567 | .597 |
| Independence model | 3.778 | 3.387 | 4.202 | 3.784 |

##### HOELTER

| Model | HOELTER .05 | HOELTER .01 |
| --- | --- | --- |
| Default model | 193 | 220 |
| Independence model | 20 | 23 |

##### Execution time summary

| Minimization: | .016 |
| --- | --- |
| Miscellaneous: | .119 |
| Bootstrap: | .000 |
| Total: | .135 |
